# Editorial to “Uncoupling endocardial bundles coupled by an epicardial bundle in the left atrium and pulmonary veins”

**DOI:** 10.1002/joa3.13066

**Published:** 2024-05-27

**Authors:** Masao Takemoto, Yoshibumi Antoku, Takuya Tsuchihashi

**Affiliations:** ^1^ Cardiovascular Centre Social Medical Corporation Steel Memorial Yawata Hospital Kitakyushu Japan

**Keywords:** ablation, atrial fibrillation, epicardial bundle, epicardial connection, pulmonary vein isolation

The recent decades have seen rapid developments in the treatment of atrial fibrillation (AF) patients, especially for the use of three‐dimensional (3D) electro‐anatomical mapping systems. To prevent initiating and maintaining AF, a complete pulmonary vein (PV) isolation (PVI) should be a target of the AF treatment. A recent report has revealed that approximately 10% of patients with AF exhibit epicardial connections (ECs) within the complete PVI lines, potentially contributing to AF recurrence.^1^ Thus, with ablation techniques, various isolation lines and focal targets using electric and anatomic approaches are deployed.

The atrial architecture is highly complex. It not only exhibits 3D arrangements of circumferentially and longitudinally orientated muscle bundles but also sudden transitions in the fiber architecture from the endocardial to epicardial layers.^2^ Such transitions may promote the unique pathological conduction properties associated with the development of atrial arrhythmias.^2^ Epicardial myofibers/bundles,^3^ such as the septopulmonary bundle, Marshal bundle, Bachmann bundle, and intercaval bundles, play a role in connecting the PV(s) and atrium on the epicardial side. These myofibers/bundles are anatomically inherited^3^ and predominantly situated near the right‐ and left‐sided PV carinas.^1^

A recent report has demonstrated that the left atrial (LA) wall thickness (LAWT) plays a crucial role in the recurrence of AF in patients undergoing ablation therapy.^4^ The LAWT varies from 1.5 to 6.5 mm^3^. Consequently, the definition of ECs may encompass true ECs preexisting prior to the PVI, alongside false ECs formed after the PVI as residual epicardial‐sided conduction related to a nontransmural lesion creation because of a thicker LAWT. While some ECs may result from the former between the right‐ or left‐sided PV carina and atrium, others could arise from the latter. Notably, the septopulmonary bundle's thickness makes it prone to a nontransmural lesion creation,^3^ potentially leading to ECs between the left PVs and LA.

A previous report demonstrated that the double‐Lasso technique using conventional circular mapping catheters did miss the nonisolation of the PV carina after a successful PVI, which was an independent predictor of AF recurrence after the PVI.^5^ More recently, it was revealed that ECs were mainly located on the carina, and employing a conventional circular mapping catheter missed 25% of ECs in comparison to a multi‐electrode mapping catheter (MEMC).^1^ This MEMC with two‐dimensional surfaces can take high‐density cardiac mapping in a whole new direction and could potentially enhance the detection of ECs, even considering their small size, as opposed to circular mapping or a point assessment with ablation catheters.^1^ A recent study showed that a precise EC detection and ablation using an MEMC could ameliorate the AF recurrence rate even in the presence of ECs.^1^ Consequently, employing an MEMC during AF ablation for LA and PV mapping and pacing appears to be an effective and feasible approach to achieving a successful PVI in patients with ECs, as described in this manuscript.^2^

This manuscript by Kobayashi et al. described an interesting case of the successful elimination of an epicardial bundle between the posterior left‐sided PV carina and LA roof (=septopulmonary bundle) without the completion of transmural conduction block on the LA roof. They could beautifully show both epicardial delayed conduction on the roof and an endocardial breakout at the posterior left‐sided PV carina in their second map, suggesting the uncoupling of the endocardial bundles coupled by an epicardial bundle between the posterior left‐sided PV carina and LA roof (=septopulmonary bundle) (supplementary movie 2).^2^ Furthermore, the site of the endocardial breakout at the posterior left‐sided PV carina in their second map showed fractionated and high‐frequency potentials on the posterior left‐sided PV carina (white arrow in figure 3A–C).^2^ Finally, a single ablation application at only that one point could achieve a complete PVI. They concluded that these characteristic fractionated and high‐frequency potentials may be a marker of an endocardial breakout site along the posterior wall isolation. Further research is needed to determine the management of AF in patients with ECs undergoing ablation therapy.

## CONFLICT OF INTEREST STATEMENT

The authors report no relationships that could be construed as a conflict of interest.

## FUNDING INFORMATION

None.

## Data Availability

The deidentified participant data will not be shared.
